# Stereotactic body radiotherapy (sbrt) in lung oligometastatic patients: role of local treatments

**DOI:** 10.1186/1748-717X-9-91

**Published:** 2014-04-02

**Authors:** Pierina Navarria, Anna Maria Ascolese, Stefano Tomatis, Luca Cozzi, Fiorenza De Rose, Pietro Mancosu, Filippo Alongi, Elena Clerici, Francesca Lobefalo, Angelo Tozzi, Giacomo Reggiori, Antonella Fogliata, Marta Scorsetti

**Affiliations:** 1 Department of Radiotherapy and Radiosurgery, Clinical Institute Humanitas Cancer Center, Rozzano(Milan), Italy; 2 Oncology Institute of Southern Switzerland, IOSI, Bellinzona, Switzerland

## Abstract

**Background:**

Data in the literature suggest the existence of oligometastatic disease, a state in which metastases are limited in number and site. Different kinds of local therapies have been used for the treatment of limited metastases and in the recent years reports on the use of Stereotactic Ablative radiotherapy (SABR) are emerging and the early results on local control are promising.

**Patients and methods:**

From October 2010 to February 2012, 76 consecutive patients for 118 lung lesions were treated. SABR was performed in case of controlled primary tumor, long-term of progression disease, exclusion of surgery, and number of metastatic sites ≤ 5. Different kinds of primary tumors were treated, the most common were lung and colon-rectal cancer. The total dose prescribed varied according to tumor site and maximum diameter. Dose prescription was 48 Gy in 4 fractions for peripheral lesions, 60 Gy in 8 fractions for central lesions and 60 Gy in 3 fractions for peripheral lesions with diameter ≤ 2 cm.

**Results:**

Dosimetric planning objectives were met for the cohort of patients with in particular V98% = 98.1 ± 3.4% for the CTV and mean lung dose of 3.7 ± 3.8 Gy. Radiological response was obtained in the vast majority of patients. The local control at 1, 2 and 3 years was 95%, 89% and 89% respectively. No major pulmonary toxicity, chest pain or rib fracture occurred. The median follow up was 20 months (range 6–45 months). Overall Survival (OS) at 1, 2 and 3 years was 84.1%, 73% and 73% respectively.

**Conclusions:**

SABR is feasible with limited morbidity and promising results in terms of local contro, survival and toxicity.

## Background

The management for patients with metastatic disease has always been focused on the use of systemic therapy alone; this potentially allows to treat microscopic metastatic tumor but generally is unable to eradicate gross tumor sites. Local therapies, although effective against single lesion tumors, have been used with palliative intent in these settings of patients. In the recent years a new evidence emerged about the existence of an intermediate condition between localized and widespread systemic disease. In 1995 Hellman and Weichselbaum first proposed the idea of an oligometastatic disease [1], in which metastases are limited in number and site. They suggested that local treatment through reduction of the tumor burden might bring to a better control of disease with consequent lengthening of lifetime. In this “scenario” local approaches, surgery or stereotactic ablative radiation therapy, have been increasingly utilized mostly for the treatment of lung and liver metastases [2,3]. Although surgery is the main modality used [4,5], more recently Stereotactic Body RadioTherapy (SBRT) is emerging as an effective alternative for patients unsuitable for surgery. This approach enables the delivery of a limited number of ablative doses with highly conformal techniques and maximum healthy tissue sparing. Retrospective and prospective reports SBRT allowed to achieve excellent local control (LC), more than 90% in some series, to delay progression, and to decrease the subsequent need of further chemotherapy treatments [5-9]. In this study a set of oligometastatic patients treated with Intensity Modulated Radiation Therapy (IMRT) using Volumetric Modulated Arc Therapy (VMAT) in the RapidArc form (RA) for lung metastases from different solid tumors was reviewed. The aim of this study was to evaluate toxicity, local control and survival.

## Materials and methods

### Patients and procedures

The present prospective study includes oligometastatic patients with lung metastases from different primary tumors. To define the appropriate therapy, each patient was evaluated by a multidisciplinary team including a radiation oncologist, a thoracic surgeon and a medical oncologist. Patient’s general condition (age, performance status) and disease status (histology, other sites of metastases, time to progression) were considered. SBRT was performed when the following criteria were met: i) controlled primary tumor, ii) absence of progressive disease longer than 6 months, iii) medically inoperable or unresectable, iv) number of metastatic sites ≤ 5 (total number in any site). No chemotherapy was given for at least 3 months preceding SBRT. The other sites of metastases outside the lung were stable or in response to previous treatment, local or systemic. From October 2010 to February 2012, 76 consecutive patients for 118 lung lesions were treated in our Department. Fifty-four were male (70%) and 22 female (30%) with a median age of 68 years (range 38–88 years). Different kinds of primary tumors were treated, the most common were lung and colon-rectal cancer. Patients characteristics, disease status and treatment features at the time of SBRT are shown in Table 1. The median time from the diagnosis to appearance of metastasis was 24 months (range, 10–144 months) and SBRT was performed at a median-time of 48 months (range, 10–180 months) after diagnosis of metastases.

**Table 1:** Patients characteristics, disease status and treatment features

**Characteristic**	**n.**	**%**
No. of patients	76	
Male	54	70
Female	22	30
68	-	
Range, years	38-88	-
Primary tumor		
Colorectal	29	38
NSCLC	18	24
Sarcoma	6	8
Genitourinary	8	10.5
Other	15	19.5
Other metastatic sites		
Bone	2	2.6
Visceral	4	5.2
Time since diagnosis (mos)		
Median	48	-
Range	10-180	-
Treatment	No.	%
Lung lesions treated	118	
No. of lung lesions (per patient)		
	49	64
	18	24
	9	12
Dose prescription (per lesion)		
48 Gy/12 Gy fraction	95	80.5
60 Gy/20 Gy fraction	7	6
60 Gy/7.5 Gy fraction	16	13.5

### Treatment

The total dose of SBRT prescribed varied according to the tumor site (central or peripheral) and maximum diameter of the lesions. For peripheral lesions up to 2 cm the dose prescription was 60 Gy in 3 consecutive fractions, for peripheral lesions between 2 and 3 cm it was 48 Gy in 4 consecutive fractions while for central lesions was 60 Gy in 8 consecutive fractions. All patients were planned and treated in supine position with arms crossed above their head. A thermoplastic mask was used to immobilize the thoracic and abdominal regions and reduce the residual body motion. A 4D-CT scan going from the mandible to the third lumbar vertebra was acquired for all patients for planning purposes. The Gross Tumor Volume (GTV) consisted of radiological lesion identified in lung parenchyma window and was delineated in each 4D-CT phase; the Clinical target volume (CTV) was defined as coincident to the GTV. The Internal Target Volume (ITV) was defined as the envelope of the CTVs from each respiratory phase. The final PTV was defined as the ITV plus an isotropic margin of 5 mm. Organs at risk (OARs) considered were: lungs, heart, spinal cord, oesophagus and chest wall. The plan objective was to cover at least 98% of the CTV (ITV) volume with 98% of the prescribed dose (V_98%_ = 98%) and for the PTV to cover 95% of the volume with 95% of the dose (V_95%_ = 95%). Constraints for OARs were D_1%_ < 20 Gy on spinal cord, D_1%_ < 30 Gy for heart and oesophagus. For ipsi- contra- and joint lungs excluding PTV, constraints of V_5Gy_ < 30%, V_10Gy_ < 20%, V_20Gy_ < 10% were set and a mean lung dose <4 Gy was imposed. All RA plans were designed and optimised with RA technique (Version 8.9-10.28, Varian, Palo Alto, CA, USA) using two partial and coplanar arcs of ≈ 200°; single isocentre was used in most of the cases. Jaw tracking was used to best reduce the leaf residual transmission. The final dose distributions were computed with the Analytical Anisotropic Algorithm (AAA) implemented in the Eclipse planning system. Patients were treated with 10MV flattening filter free (FFF) beams using the TrueBeam linac (Varian, Palo Alto, CA, USA) equipped with the millennium MLC with leaf dimension at isocenter of 5 mm. A maximum dose rate of 2400 MU/min was used. Before each fraction image guided RT was performed by means of kV cone beam CT (CBCT) aligning to the tumor in the reference planning CT.

### Outcome evaluation

Clinical outcome was evaluated by thoracic and abdominal CT scan and 18FDG-PET-CT before treatment, CT scan every 3 months and/or 18FDG-PET-CT if needed after treatment. Complete Remission (CR) was defined as the disappearance of the lesions at CT scan; a reduction greater than 30% was considered Partial Remission (PR); any growing lesion not clearly ascribable to fibrosis was reported as Progression of Disease (PD). The incidence of toxicity was graded according to the NCI CTCAE vs 3.0 scale every 3 months as well.

### Statistical analysis

Data are reported as number and percentage or mean and SD where appropriate. Survival curves were generated using the Kaplan-Meier method. Overall survival times were calculated starting from the treatment beginning. All analyses were performed with Stata 10 (StataCorp, College Station, TX).

## Results

**Figure 1:**
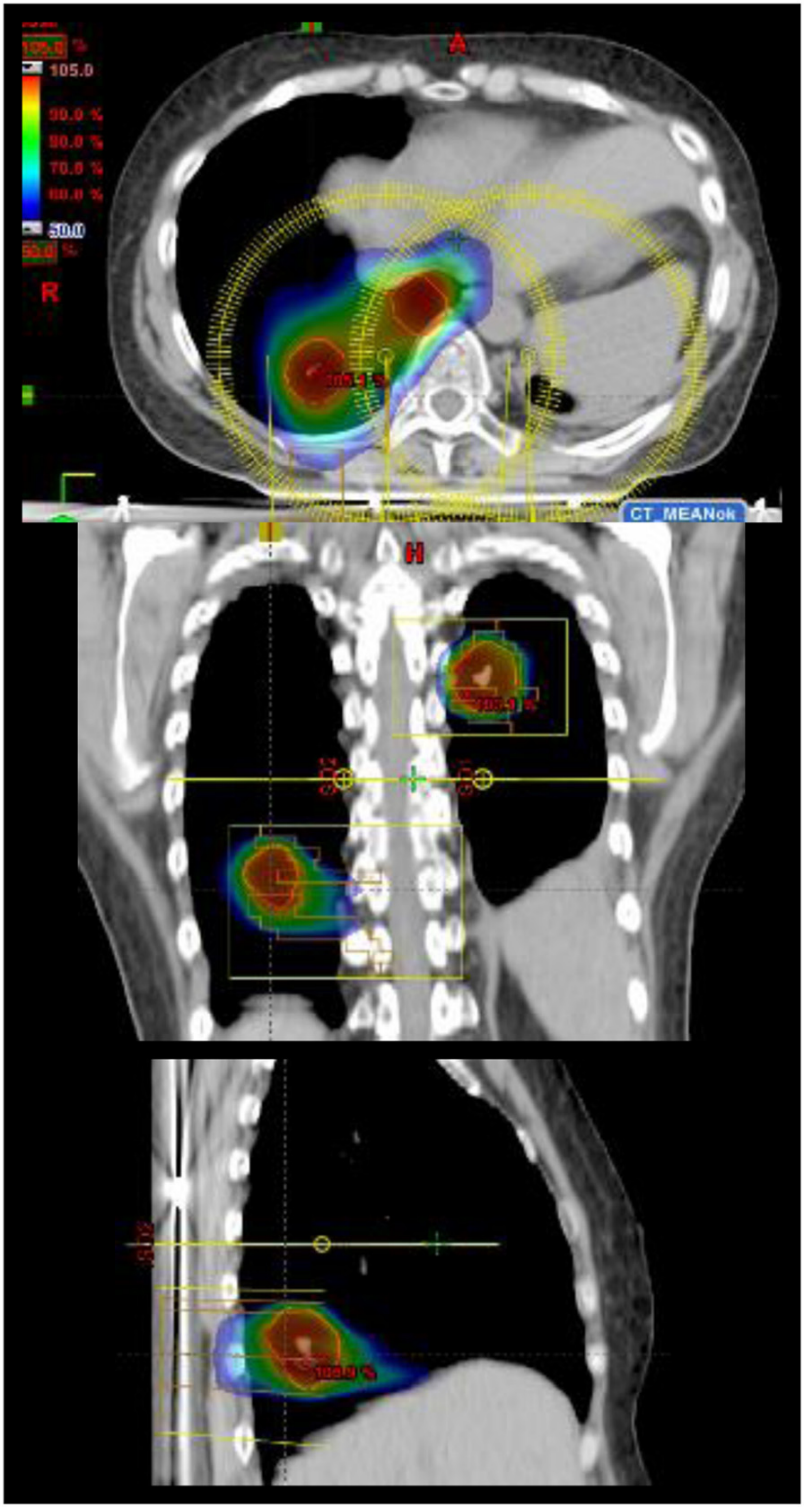
**An example of dose distribution for a 3-lesion patient.** The 50% isodose distribution is pointed out showing a qualitative overview of the target coverage and dose fall down outside the PTV.

In Figure 1, an example of dose distribution in one of our patients is shown, providing a qualitative overview of the target coverage and dose fall off outside the PTV. Similar results were obtained for the other patients.

A total of 118 lesions were treated in 76 patients. In 27/76 patients (36%) more than one lung metastases was irradiated. Dose prescription varied according to lesions site, peripheral or central, and volume: 48 Gy/12 Gy fraction in 95 lesions (80.5%); 60 Gy/7.5 Gy fraction in 16 lesions (13.5%), and 60 Gy/20 Gy fraction in 7 lesions (6%) as shown in Table 1. All patients completed the planned treatment. The 6 patients in the cohort who were affected by synchronous metastases in the lung and in other sites, were treated only for the lung lesions because the disease in the other sites (bones and viscera) was stable and/or asymptomatic.

### Dosimetric data

**Figure 2:**
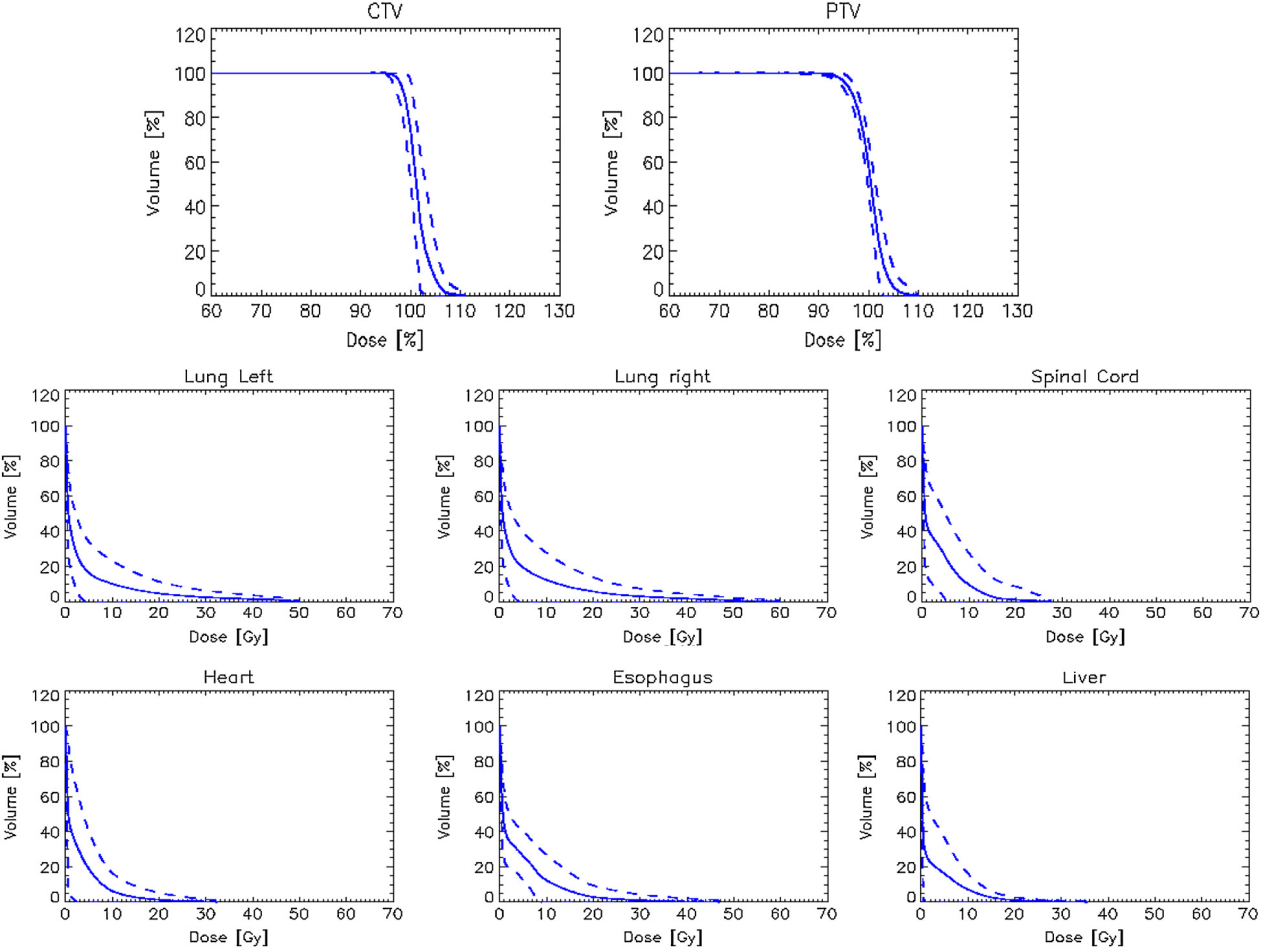
**Average dose volume histograms for CTV, PTV and organs at risk.** Dashed lines represent inter-patient variability at + -1 standard deviation.

Figure 1 presents an example of dose distributions in axial, coronal and sagittal views for one patient for one of the most complex patients with two isocentres. Color wash for dose scaling was set in the range 50-105%. Figure 2 shows the average cumulative dose volume histograms (DVH) computed for the whole cohort of patients (solid lines). The dashed lines represent the inter-patient variability expressed at + -1 standard deviation. For CTV and PTV, given the different prescriptions, dose is expressed in percentage. For Organs At risk in Gy. Table 2 summarised the numerical analysis performed on CTV, PTV and OARs and based on DVHs. Reported are the main parameters valuable for plan assessment, the corresponding planning objectives, the mean values of the findings (with 1 standard deviation uncertainty) and the observed range. As it can be derived from the table, all objectives were on average met.

**Table 2:** Summary of the DVH analysis for the CTV, PTV and Organs at Risk

**Parameter**	**Objective**	**Mean ± SD**	**Range**
**CTV**			
Volume = 13.9 ± 25.8 cm^3^ Range = [.5 – 183.6] cm^3^			
**Mean [Gy]**	100%	101.4 ± 1.7	[98.4 - 106.4]
**V**_**98% **_**[%]**	>98%	98.1 ± 3.4	[80.3 - 100.0]
**D**_**99% **_**[Gy]**	Maximize	98.25 ± 1.7	[94.3 - 103.2]
**D**_**1% **_**[Gy]**	Minimize	104.3 ± 2.1	[99.8 - 112.9]
**PTV**			
Volume = 42.3 ± 51.2 cm^3^ Range = [2.5 – 333.1] cm^3^			
**Mean [Gy]**	100%	100.3 ± 0.3	[96.8 - 104.9]
**V**_**95% **_**[%]**	>95%	96.7 ± 1.9	[90.2 - 99.9]
**D**_**99% **_**[Gy]**	Maximize	93.1 ± 2.0	[89.5 - 96.6]
**D**_**1% **_**[Gy]**	Minimize	104.8 ± 1.8	[101.9 - 112.9]
**Lungs**			
**Mean [Gy]**	<4 Gy	3.7 ± 3.8	[0.5 - 16.8]
**V**_**5Gy **_**[%]**	<30%	17.9 ± 18.8	[0.1 - 78.2]
**V**_**10Gy **_**[%]**	<20%	11.2 ± 14.2	[0.1 - 65.8]
**V**_**20Gy **_**[%]**	<10%	5.2 ± 7.4	[0.0 - 34.6]
**Spinal cord**			
**D**_**1% **_**[Gy]**	<20 Gy	10.2 ± 5.6	[2.2 - 21.7]
**Heart**			
**D**_**1% **_**[Gy]**	<30 Gy	11.3 ± 10.9	[0.5 - 28.6]
**Esophagus**			
**D**_**1% **_**[Gy]**	<30 Gy	16.3 ± 11.5	[5.1 - 36.1]

Dx%: dose received by at least x% of the volume; Vx%: volume receiving at least x% of the dose.

Data are reported as average values plus or minus standard deviation and range.

### Local control

**Figure 3:**
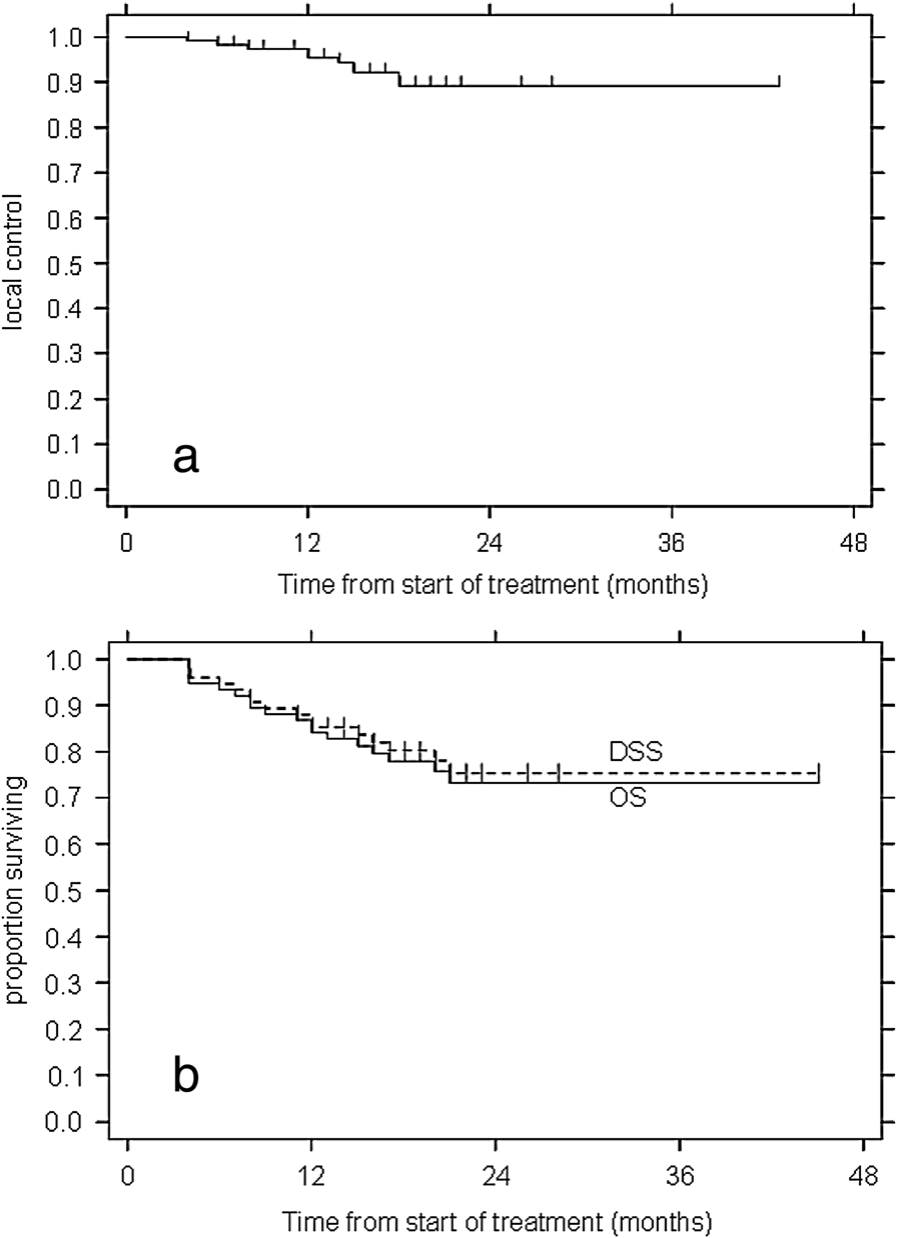
**a) Local control; b) Overall survival (OS) and Disease Specific Survival (DSS).** Figure a refers to the 118 treated lesions while b to the 76 patients.

A radiological response was obtained in all lesions after treatment. At the last observation, Complete Remission (CR) was recorded in 71/118 cases (60%) Partial Remission (PR) in 33/118 (28.5%), Stable disease (SD) in 4/118 (3%) and in-field-progression in 10/118 (8.5%). About in field progression it was exclusively local in 5 cases (3 patients) and occurred at 6, 12 and 18 months after treatment; patients underwent to new line of chemotherapy and are alive at 21, 22 and 23 months. In the other 5 cases (4 patients) it was associated with distant tumor progression and 3/4 patients died within a year from the progression. The 1, 2 and 3 years Local Control Rate was 95%, 89% and 89% respectively as shown in Figure 3a. Depending on the primary tumor, recurrence appeared in 3 of 29 patients with colorectal tumor (locally and distance), and in 4 of 15 patients with other histology (2 locally and distance). No correlation between delivered doses and local control was present. The median time between SBRT and progression (and therefore new chemotherapy treatment) was 10 months (range: 3–19 months).

### Toxicity

No acute toxicity occurred. At the last examination 80% of patients presented a late G1 lung toxicity (mostly radiological fibrosis in <25% of lung volume. No severe (G2-G4) pulmonary toxicity, chest pain or rib fracture was observed until last examination.

### Survival

The median follow up was 18 months (range 6–45 months). At the time of analysis 58 patients (76%) are alive and 18 (24%) patients are dead; 2 patients died early for causes unrelated to disease progression or treatment toxicity. The median survival was 20 months (range 6–45 months). OS at 1, 2 and 3 years was 84% , 73% and 73% respectively; Disease Specific Survival (DSS) at 1,2 and 3 years was 85%, 75% and 75% respectively (Figure 3b). Progression free survival (PFS) at 1,2 and 3 years was 83%, 70% and 70% respectively. In univariate analisis, no correlation was found between survival and sex, age, presence and site of other sites of metastases, volume of lung lesions or total dose of SBRT delivered. Neither the histology of primary tumor, affected survival: 2-year OS in patients with lung metastases from Colorectal tumor , NSCLC and Genitourinary tract was 78%, 77% and 75% respectively. Only in patients with sarcoma the 2 years OS was 100% but the number of patients is very low (6 patients).

## Discussion

The treatment for metastatic patients mainly consisted in the use of chemotherapeutic agents; the median survival and the treatment choice depend on several prognostic factors: patients age, PS, primary histology, site and number of metastases. The role of radiation therapy in the management of metastatic disease has been principally limited to palliation but we now have an opportunity to challenge this statement. Probably there is an intermediate state defined by a long latent interval between the treatment of the primary tumour and the appearance of metastases. Hellman and Weichselbaum [1] first proposed the idea of an oligometastatic state in 1995. They suggested that for many cancers a few metastases exist at first, before the malignant cells acquire widespread metastatic potential. Theoretically, if ablative hypofractionated radiation therapy could be delivered during the oligometastatic phase, this might modify disease outcome in patients in which up to now radiotherapy was used only with a palliative intent. The role of SBRT for the treatment of oligometastases was investigated also in other sites, often localized in not-accessible sites, Casamassima [10] investigated SBRT in adrenal gland metastases showing in a retrospective analysis on 48 patients an actuarial local control as great as 90%. Bonomo [11] investigated the role of SBRT in paracardiac metastases (on 16 patients) and demonstrated a crude local control of 75% in this challenging localisation. Experimental data from multiple cancer models have provided sufficient evidence to propose a paradigm shift, since some of the effects of ionizing radiation are recognized to give a contribution to systemic antitumor immunity. Recent examples of objective responses achieved by adding radiotherapy to immunotherapy in metastatic cancer patients support this view. Therefore, the traditional palliative role of radiotherapy in metastatic disease is evolving into that of a powerful adjuvant for immunotherapy [12]. Innovative techniques have revolutionized radiation oncology within the last decade. Using IMRT or VMAT the conformity of the dose to the tumor is increased while sparing of healthy tissue is optimized. More recently, in our experience, RA with FFF beams permits a best treatment. In our series oligometastatic patients with lung metastases from different kind of solid tumors underwent SBRT with RA. Using this approach good results were obtained in terms of local response in most patients, local control rate at 1, 2 and 3 years was 95%, 89% and 89% respectively. These results are comparable with other studies in literature. In Table 3 selected trials of stereotactic body radiation therapy for lung metastases are shown [13-18]. Le [14] reported the results of a phase II trial using SBRT with a dose of 50 Gy in 10 fractions in the treatment of oligometastatic disease resulting in a 2-year local control rate for all treated lesions of 67%. Several recent reports recorded high rate of OS using SBRT in oligometastatic patients. Norihisa [15] treated 35 patients with one or two lung metastases. The primary site was controlled and there were no other organs involved. The original dose of 48 Gy in four fractions was escalated to 60 Gy in five fractions for 16 patients. Two-year results for overall survival, local control and progression-free survival were 84%, 90% and 35%, respectively. Again, Salama [16] treated 61 patients with 113 metastases performing a dose escalation increasing from 24 Gy in 3 fractions to 48 Gy in 3 fractions. One and 2 year progression free survival were 33.3% and 22%, 1-year and 2-year overall survival were 81.5% and 56.7%. Finally, Rusthoven [17] reported a phase I/II prospective study of SBRT for metastatic lung tumors. Thirty-eight patients with 63 lesions were treated and achieved a 2-year local control rate of 96%, but a 2-year overall survival of only 39%. Also in terms of OS at 1 and 2 years our results are comparable with these series with values of 85% and 66% respectively. Despite the heterogeneous characteristics of oligometastatic patients present both in our data and in other published studies, these findings indicate that the use of SBRT gives an advantage in terms of local control, disease free survival and overall survival to a significant percentage of patients: generally ~ 20% of patients remain disease-free 2–4 years after SBRT [13-23]. Generally SBRT presents negligible toxicity: the ratio of patients with grade 3 acute or late adverse event is less than 10%; in our series no grade 3 or late toxicity was recorded. Different prognostic factors were analysed, in particular age, sex, primary tumor histology, disease free interval, metastases site, size and number and dose prescription. As shown in several series the following factors significantly affect local control, PFS and OS: some histology of primary tumor (i.e. breast cancer) [24], disease-free interval of more than 12 months [25,26], target position [17,27-29], number (≤3) [16,30-32] and size of metastases (smaller than 3 cm) [17,20,21,33-35], and total delivered dose with biological equivalent dose (BED) larger than 100 Gy_10_[24-37]. In our experience, however, no factors statistically affected outcome of patients. This finding is probably related to the characteristics of our series: most patients had unfavourable histology (mostly GI tumor and NSCLC), all had primary tumor controlled with disease free interval greater than 12 months.

**Table 3:** Summary of selected trials of stereotactic body radiation therapy for lung metastases

**Study**	**No patients**	**Median dose/no fractions**	**Median FU mos (range)**	**Local control rate**	**Overall survival**	**Toxicity**
Okunieff et al [13]	50	50 Gy/10	18.7 (3.7-60.9)	3-yr 91%	2-yr 50%	G2 6.1%
		48 Gy/6				G3 2%
		57 Gy/3				
Norihisa et al [15]	34	48 Gy/4	27 (10-80)	2-yr 90%	2-yr 84%	G2 12%
		60 Gy/5				G3 3%
Le et al [14]	32	15 Gy/1	-	1-yr 54%		G2-G3 4
		20 Gy/1		1-yr 54%		G5 3
		25 Gy/1		1-yr 91%		Dose ≥ 25 Gy
		30 Gy/1		1-yr 91%		
				2-yr 67%		
Salama et al [16]	61	24 Gy/3	20.9 (3-60.5)	1-yr 67.2%	2-yr 56.7%	G3 3
		48 Gy/3		2-yr 52.7%		
Rushoven et al [17]	38	60 Gy/3	15.4 (6-48)	2-yr 96%	2-yr 39%	G3 3 (8%)
Ricardi et al [18]	61	45 Gy/3	20.4 (3-77)	2-yr 89%	2-yr 66.5%	G3 1 (1.6%)
		26 Gy/1				

## Conclusion

We have shown that, in oligometastatic patients with lung metastases, SBRT is feasible with limited morbidity. The clinical results obtained are highly satisfactory. Well-designed collaborative trials, including stratification of patients by histology, are necessary to draw final conclusions, in a multidisciplinary perspective, in this field and to determine whether SABR, with it’s lower morbidity, could be a real alternative to surgery in the treatment of oligometastatic patients.

## Competing interests

Dr. L. Cozzi acts as Scientific Advisor to Varian Medical Systems and is Head of Research and Technological Development to Oncology Institute of Southern Switzerland, IOSI, Bellinzona. The study was partially financed by a research grant of Varian Medical systems. Other Conflict of Interest: None.

## Authors’ contributions

PN, MS, FA, LC designed the study and the analysis. AMA, FDR, EC, AT collected the clinical data, PM, ST, FL, GR collected the dosimetric data. AFC, LC, PN, AMA performed main data analysis. PN, LC drafted the manuscript. All authors reviewed and approved the final manuscript
